# The influence of dietary and whole-body nutrient content on the excretion of a vertebrate consumer

**DOI:** 10.1371/journal.pone.0187931

**Published:** 2017-11-27

**Authors:** Christopher M. Dalton, Rana W. El-Sabaawi, Dale C. Honeyfield, Sonya K. Auer, David N. Reznick, Alexander S. Flecker

**Affiliations:** 1 Department of Ecology and Evolutionary Biology, Cornell University, Ithaca, New York, United States of America; 2 Department of Biology, University of Victoria, Victoria British Columbia, Canada; 3 Northern Appalachian Research Lab, United States Geological Survey, Wellsboro, Pennsylvania, United States of America; 4 Institute of Biodiversity, Animal Health and Comparative Medicine, University of Glasgow, Glasgow, United Kingdom; 5 Department of Biology, University of California Riverside, Riverside, California, United States of America; Stockholm University, SWEDEN

## Abstract

In many contexts, nutrient excretion by consumers can impact ecosystems by altering the availability of limiting nutrients. Variation in nutrient excretion can be predicted by mass balance models, most of which are premised on two key ideas: (1) consumers maintain fixed whole-body nutrient content (*i*.*e*., %N and %P), so-called *fixed homeostasis*; (2) if dietary nutrients are not matched to whole-body nutrients, excesses of any nutrient are released as excretion to maintain fixed homeostasis. Mass balance models thus predict that consumer excretion should be positively correlated with diet nutrients and negatively correlated with whole-body nutrients. Recent meta-analyses and field studies, however, have often failed to find these expected patterns, potentially because of a confounding influence—flexibility in whole-body nutrient content with diet quality (flexible homeostasis). Here, we explore the impact of flexible homeostasis on nutrient excretion by comparing the N and P excretion of four genetically diverged Trinidadian guppy (*Poecilia reticulata*) populations when reared on diets of variable P content. As predicted by mass balance, P excretion increased on the high-P diet, but, contrary to the notion of fixed homeostasis, guppy whole-body %P also increased on the high-P diet. While there was no overall correlation between excretion nutrients and whole-body nutrients, when the effect of diet on both whole-body and excretion nutrients was included, we detected the expected negative correlation between whole-body N:P and excretion N:P. This last result suggests that mass balance can predict excretion rates within species, but only if dietary effects on whole-body nutrient content are controlled. Flexible homeostasis can obscure patterns predicted by mass balance, creating an imperative to accurately capture an organism’s diet quality in predicting its excretion rate.

## Introduction

Consumers are a critical component of nutrient cycles, and variation in the rate at which consumers excrete dissolved nutrients, like nitrogen (N) and phosphorus (P), can alter nutrient limitation and ecosystem function [1–3]. Because variation in excretion can affect ecosystems, ecologists have sought to understand the causes of variation in excretion rates, often using the principle of mass-balance [4,5]. Most models employing mass balance assume that each consumer has an immutable whole-body nutrient content, determined by its genetics, sex, and ontogeny [6–8]. By these models, all consumers maintain homeostasis by precisely regulating their whole-body %N and %P–retaining the nutrients needed to build tissue and releasing excess nutrients as waste, often as excretion. Mass balance models thus produce two simple predictions about consumer excretion: (1) a consumer with a high-nutrient diet will excrete more nutrient; (2) a consumer with high-nutrient tissues will retain more nutrient and thus excrete less. Yet, while some field studies have found evidence for these patterns [9–11], many, especially those within a single species, have failed to do so [9,12–14].

This failure may stem from various sources, such as variation in assimilation efficiency [15] or flexibility in consumers’ whole-body nutrient content to diet quality. Recent empirical studies have shown that the assumption of an immutable whole-body nutrient content, or fixed homeostasis, is often violated [16,17]. For example, vertebrate consumers can retain excess dietary nutrients in flexible storage pools of carbon (in fat) and phosphorus (in bone) [18–20]. This ‘flexible homeostasis’ leads to the prediction that both whole-body and excretion nutrients should increase on a high-nutrient diet, yielding a positive correlation. This positive correlation, however, would counter the negative correlation predicted by mass balance. Isolating the effects of fixed homeostasis (as determined by phenotype) and flexible homeostasis (as determined by diet quality) is thus central to whether mass balance can predict variation in consumer excretion. Here, we probe the role of genetics and diet quality in whole-body and excretion nutrients using a model for intraspecific trait variation: the Trinidadian guppy (*Poecilia reticulata*).

Within Trinidadian rivers, guppies live in environments that vary greatly in their degree of predation risk [21] and available resources [22]. In turn, guppies show extensive variation in their diet nutrient content, whole-body nutrient content, and nutrient excretion rates [23–26]. In their ancestral environments, which have high-predation risk (HPred), guppies exploit abundant, nutrient-rich invertebrate foods [23,26]. In derived environments with low predation risk (LPred), guppies consume low-nutrient foods and have evolved altered life history traits [23,26,27]. HPred guppies excrete N and P at higher rates than LPred guppies [25,28,29], possibly because of their higher nutrient diets [23,26] or genetic differences in traits that affect excretion, such as whole-body nutrient content and metabolism [23,30]. Guppies thus present an excellent system for studying how intraspecific variation in nutrient excretion may reflect environmental effects (*i*.*e*., diet quality) or evolution (*i*.*e*., genetic divergence).

In this study, we compared the whole-body and excretion nutrients of guppies from four genetically diverged populations, to assess the influence of genetic divergence on these traits. Within each population, we also systematically varied the diet P content, to assess the influence of diet quality on whole-body and excretion nutrients. Our results enable us to independently assess the roles of diet quality and genetic divergence on whole-body and excretion nutrients, to provide insight on the ability for mass-balance models to effectively explain variation in excretion.

## Materials and methods

### Lab study design

We bred guppies from two rivers in Trinidad (Aripo and Guanapo Rivers) under standardized lab conditions to provide subjects for the experiment. Within each river, we collected guppies in 2009 from an HPred locality where guppies coexist with a diversity of large piscivorous fish, and an LPred locality without piscivorous fish. HPred and LPred guppy populations within each of these two drainages are known to have diverged genetically in life history traits [31,32]. Wild-caught females from each population were kept individually, either in 20 L glass tanks with box filters or in 3 L tanks in recirculating systems (12L:12D, temperature 25 ± 1°C) and fed twice daily (AM: Tetramin^TM^ tropical fish flake paste, PM: hatched immature *Artemia* spp.). All experiments were carried out at Cornell University. Guppies from the Guanapo populations were first lab-born generation guppies, and those from the Aripo populations were second lab-born generation guppies. Experiments were conducted from September–December 2010, with all experiments occurring simultaneously in that time period.

Fourteen juvenile female guppies from Aripo HPred and LPred, all between 24–35 days old, were randomly assigned to one of two treatment diets (n = 7 per predation history × diet treatment). Eighteen adult female guppies from Guanapo HPred and LPred were randomly assigned to two treatment diets (n = 9 per predation history × diet treatment). Aripo guppies were stocked singly into 1.5 L tanks on a recirculating zebrafish system (Aquatic Habitats, Apopka, FL) with collective filtration. Guanapo guppies housed in groups of three in 20 L tanks filled with 19 L of water with physical and carbon filtration. Differences among the two rivers (Guanapo vs. Aripo) in the age of the fish and rearing conditions were due to logistical constraints at the time of the experiment. These differences do not inhibit the ability to detect genetic divergence or plasticity within each river. Tanks were randomly assigned to locations across shelving units. Water for all guppies was provided from a central source tank that contained deionized water, Instant Ocean ®, and sodium bicarbonate (Instant Ocean ®: 0.88 g L^-1^; Sodium Bicarbonate: 0.14 g L^-1^). This formulation provided water with pH, general hardness, and carbonate hardness comparable to Trinidadian streams.

For 14 weeks (Aripo) and 11 weeks (Guanapo), guppies were fed one of two standardized treatment rations twice daily. Treatment diets consisted of either a low %P or a high %P and were designed following diet specifications in Shim and Ho[33]. A formulation for this diet can be found in S1 Table. Both diets were replete in energy and all other macro and micro nutrients. High and low-P diets were identical aside from the amount of added inorganic P. The high-P diet had % P comparable to a high-quality invertebrate diet (0.81% of dry weight in P), while the low-P diet was comparable to a diet mixing some invertebrates with detritus and algae (0.26% P; Shim & Ho 1989). The quantities of diet fed were aligned to be isocaloric with those in the protocols of previous researchers [34] and were never lower than 7% of body weight per day. Dried food was kept frozen until used. Each day, it was mixed with water at a 3:2 food:water ratio, and it was delivered using a Hamilton luer-tip microsyringe. Uneaten food was rarely noted in any tank and, when present, was removed from each tank at the end of the day using a plastic pipette.

### Response variables

The post-feeding excretion rate of each fish was measured on the last day of the experiment. After allowing guppies 60 minutes to consume a normal single feeding’s ration, we removed guppies from their experimental tanks, introduced them into plastic beakers containing 250 mL of filtered tank water (GF/F with pore size = 0.7 μm, Whatman), and placed in an opaque shelter to minimize disturbance during the incubation. After 20 minutes, we collected a water sample from each beaker using a 60-mL plastic syringe. We collected a second water sample from each beaker after another 60 minutes. Prior trials revealed that 20 minutes of acclimation was sufficient to alleviate handling stress, and 60-minute incubations minimized fasting effects [35]. The timing of excretion rates was based on previous pilot studies, which showed guppies maintain fairly consistent post-prandial excretion rates for at least 4 hours post-consumption.

Subsamples for soluble reactive phosphorus (SRP) and ammonia (NH_3_) analysis were filtered through previously ashed glass-fiber filters (GF/F Whatman, pore size = 0.7 μm), refrigerated within 20 minutes of collection, and analyzed within 12 hours. NH_3_ concentrations were measured on an Aquafluor handheld fluorometer (Turner Designs, Sunnyvale, CA, USA), equipped with a UV filter [36,37], with a method detection limit of 0.2 μg N L^-1^. SRP was determined using the molybdate method [38,39]. Samples were measured on a Shimadzu UV mini 1240 spectrophotometer (Shimadzu Scientific Instruments, Columbia, MD, USA), with a method detection limit of 1.25 μg L^-1^, though analysis of standard samples repeatedly detected P at 0.5 μg L^-1^. Hourly excretion was estimated as the difference in nutrient concentration between the two samples divided by the length of the incubation, in hours, after correcting for the volume of water in the beaker during the incubation. Controls used in an initial pilot study never exceeded 5% of fish excretions and were not performed for laboratory measurements.

At the conclusion of the excretion measurement, guppies were measured for length and weight, sacrificed using MS-222, placed on ice, and frozen to -10°C within 1 hour. As in previous studies of guppy whole-body stoichiometry [23], guppy digestive tracts were dissected and discarded prior to whole-body nutrient analysis, and the combined somatic and reproductive tissues of each guppy were dried to constant mass at 55°C. Dried guppies were then weighed and ground into a homogeneous powder using a Wig-L Bug® tissue grinder.

Subsamples (2 mg for C and N, 20–40 mg for P) of guppy tissue were then assayed for C, N and P content following methods described in El-Sabaawi et al [23]. Briefly, 2 mg subsamples of homogenized tissue were weighed to the nearest 0.001 mg, and the percent C and N of each sample was assayed (Vario EL III elemental analyzer, Elementar, Hanau Germany). The remainder of the guppy tissue was weighed, ashed in Pyrex vials at 500°C for 2 h and digested in 1N HCl at 105°C to facilitate dissolution of P. SRP of the resulting solution was then quantified using serial dilutions and the molybdate blue method. Spinach and bone meal standards NIST SRM 1486 and SRM 1570a were used as known reference points in the analysis to ensure complete digestion and accurate estimates of tissue P content.

### Statistical analysis

All data were analyzed in R [40] using model comparison. The influences of diet P content and ancestral predation environment (HPred vs. LPred) on response variables were the main focus of our analysis and were included as fixed effects in models. Differences between Aripo and Guanapo guppy populations, which also reflect differences in rearing environment and fish age, were accounted for by including ‘River’ as a random effect in each model. We also analyzed each river independently, and those results are presented in the supplemental materials (S3 Table).

The most complex, biologically-feasible models were simplified to the best fit models using likelihood-ratio tests and corrected Akaike Information Criteria (AICc) score comparison [41]. N and P excretion rates, which scale allometrically with weight, were corrected for the expected ¾ power scaling of metabolism with weight by dividing measured excretion by fish weight raised to the ¾ power [10,42]. Species vary substantially in their allometric scaling coefficients [43], but we chose to use this general model since size was confounded with some of our population differences. All models assume a Gaussian distribution and were fitted using the lme4 package in R [44]. The suitability of data variance distributions to these analytic methods was validated using the R package *Global Validation of Linear Model Assumptions* [45] and by visual inspection of plots of variance distributions and residuals.

Because guppies in the Guanapo were reared in groups of three, the three guppies in each tank were non-independent. We tested the hypothesis that guppies reared in the same tank were more similar to each other than to other guppies by including tank as a random effect in each model. Such “tank effects” never increased model explanatory power, (i.e., ∆AIC ~ 2), indicating rearing guppies in group tanks was not appreciably different from rearing them in isolation. “Tank” effects were therefore not included in the analyses reported below.

### Ethical statement

Research was conducted under Cornell University IACUC Protocol 2008–0106. Research animals were collected using fine-mesh dip nets, and, when necessary, humanely sacrificed using an overdose of MS-222, in accordance with the Cornell University IACUC-approved protocol related to this research. The Cornell University Institutional Care and Use Committee (IACUC) specifically approved this study. No endangered or protected species were used in this research.

Fish collection and export was approved by Ministry of Agriculture, Land and Marine Resources, Republic of Trinidad and Tobago, conforming to their legislation. Ministry officials were made aware of field sampling and laboratory research methods prior to collection. Guppies collected at the Guanapo High Predation site were made with permission of the landowner, Tunapuna/Piarco Municipal Corporation. No further specific permission was required from field sites, which were made from public roadsides with specific permission of the Ministry of Agriculture, Land and Marine Resources, Republic of Trinidad and Tobago.

## Results

### Whole-body nutrient content

**Fig 1:**
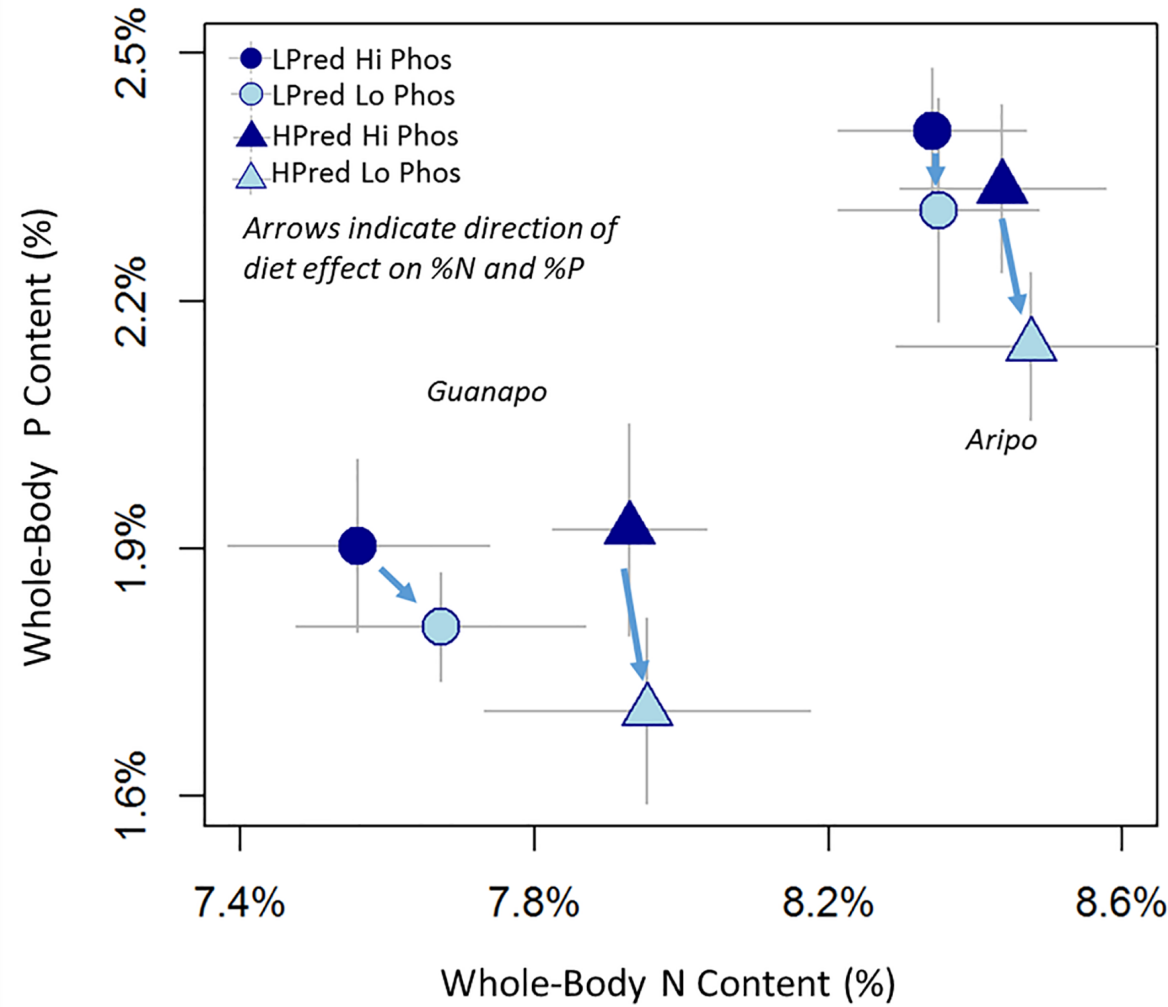
Population mean whole-body %P vs. whole-body %N for HPred (triangles) and LPred (circles) on the high-P diet (dark blue) and low-P diet (light blue), with standard errors represented in light gray lines. Symbols on the left correspond to the Guanapo guppies, and those on the right correspond to the Aripo guppies. Low-P diets reduced whole-body %P, as indicated by blue arrows. HPred guppies in both rivers had higher average whole-body N than LPred guppies (triangles vs. circles). On average, Guanapo guppies are lower %N and %P because they are higher %C.

The low-P diet weakly increased whole-body C (p = 0.09), decreased whole-body P (p = 0.04) and did not affect whole-body N (Fig 1; Table A in S2 Table and accompanying text). Ancestral predation environment was weakly associated with whole-body N (p = 0.05), as guppies from HP sites had marginally higher whole-body N than those from LP sites. In general, the magnitudes of predation and diet quality effects were small (Table A in S2 Table and accompanying text), though high-P diet guppies averaged 12% higher whole-body P than low-P diet guppies (2.2% vs. 2.0%).

The low-P diet increased whole-body molar ratios of C:P (p = 0.03) and N:P (p = 0.04) and did not affect whole-body C:N (Table B in S2 Table). Guppies on the low-P diet had 9% higher C:P and 8% higher N:P than guppies on the high-P diet, reflecting less whole-body P. HPred guppies averaged lower whole-body C:N than LPred guppies, though this effect was not significant (p = 0.16 Table B in S2 Table). HPred guppies had 3% lower whole-body C:N than LPred guppies (HPred C:N = 7.4; LPred C:N = 7.6).

### N and P excretion

**Fig 2:**
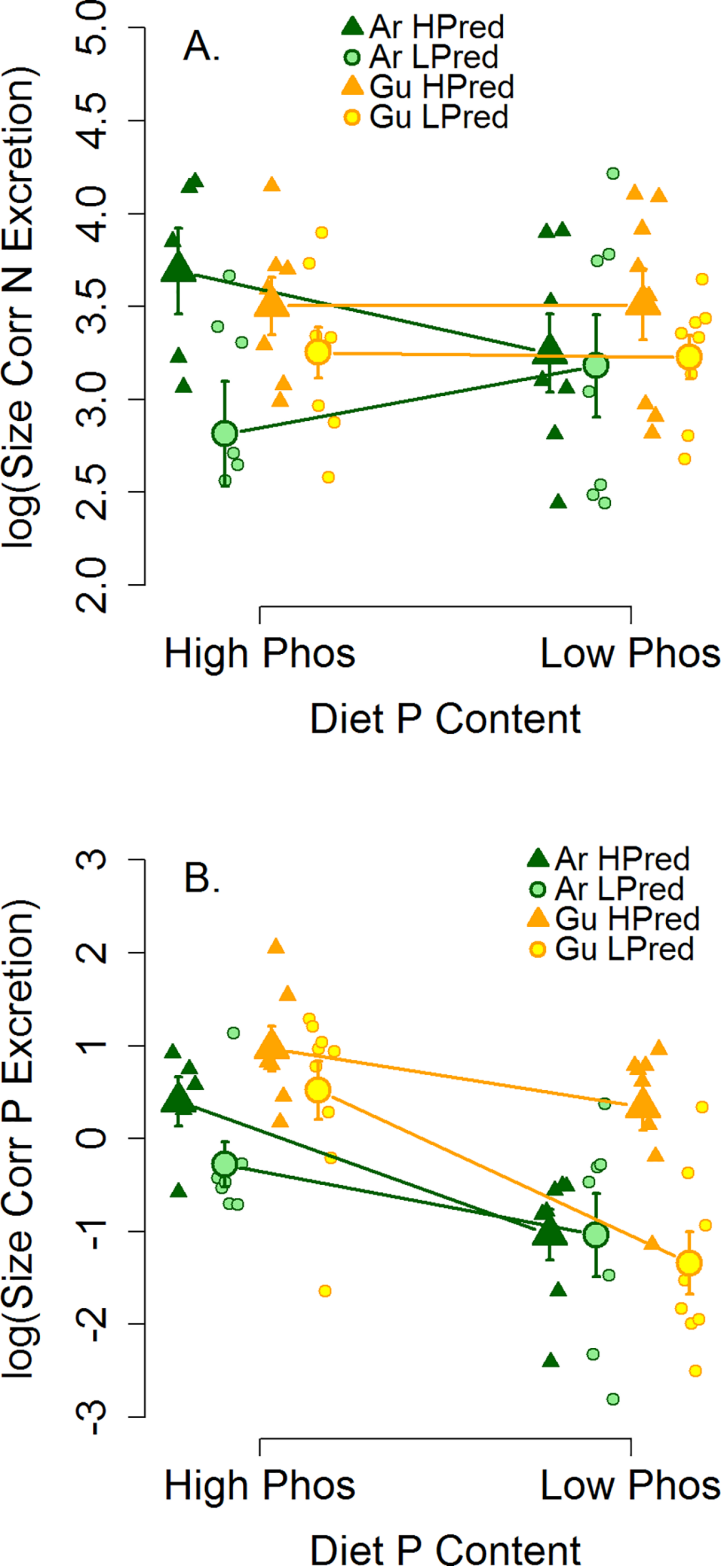
P Excretion (A) and N excretion (C) for the Guanapo (yellow) and Aripo (green) rivers. Size correction was conducted by dividing measured excretion by the fish’s weight, raised to the ¾ power to account for expected metabolic scaling. Low-P diet guppies had lower P excretion in both the Guanapo and Aripo. Both P excretion and N excretion were higher in HPred populations (orange) than LPred (blue), though this effect was only marginal in the Aripo for N excretion. Small differences in the x-axis placement within the High-P and Low-P diet groups do not reflect differences in diet quality, but are used to create separation to facilitate visual comparison of the data points.

N excretion was affected only by ancestral predation environment, while P excretion was affected by both ancestral predation environment and diet (Fig 2; Table 1). LPred guppies excreted 28% less N than HPred guppies (26 μg N hr^-1^ g ^-3/4^ vs. 36 μg N hr^-1^ g ^-3/4^). Guppies on the low-P diet excreted 62% less P than guppies on the high-P diet (0.78 μg P hr^-1^ g ^-3/4^ vs. 2.0 μg P hr^-1^ g ^-3/4^), and LPred guppies excreted 38% less P than HPred guppies (1.1 μg P hr^-1^ g ^-3/4^ vs. 1.7 μg P hr^-1^ g ^-3/4^). Excretion N:P was affected by diet and predation. Guppies on the low-P diet had a 4.8× higher mean excretion N:P (267 for low-P vs. 56 for high P) and LPred guppies had a 2.3× higher excretion N:P than HPred guppies, reflecting their substantially lower excretion P.

**Table 1:** Models of N excretion (A; size-corrected and log-transformed), P excretion (B; size corrected and log-transformed), and excretion N:P (C; log-transformed). All models include ‘river’ as a random effect, to account for differences between guppies from the Aripo and Guanapo Rivers in age, rearing environment, and background genetics. Variation in P excretion and excretion N:P suggests independent influences of diet and ancestral predation, while variation in N excretion suggests only an influence of predation. Model support for predation effects on N excretion was strong (removing predation LRT: df = 1, χ^2^ = 6.68, p = 0.01). Model support for diet and predation effects on P excretion was also strong (removing predation LRT: df = 1, χ^2^ = 10.2, p = 0.001; removing diet LRT: df = 1, χ^2^ = 22.7, p < 0.001). Model support for diet effects on excretion N:P was strong (LRT: df = 1, χ^2^ = 19.0, p < 0.001) but support for predation effects on excretion N:P was weak (LRT: df = 1, χ^2^ = 2.35, p = 0.13).

A—Models for N Excretion	AICc	∆AICc	Rel. Lik.	w_*i*_	r^2^
No effects	102.0	4.3	0.00	0.00	0.00
Diet	104.3	6.6	0.00	0.00	0.00
**Predation**	**97.7**	**0.0**	**1.00**	**0.99**	**0.11**
Diet + Pred	100.1	2.4	0.01	0.01	0.11
B—Models for P Excretion	AICc	∆AICc	Rel. Lik.	w_*i*_	r^2^
No effects	183.4	23.7	0.00	0.00	0.04
Diet	167.5	7.8	0.00	0.00	0.30
Predation	180.0	20.3	0.00	0.00	0.13
**Diet + Pred**	**159.7**	**0.0**	**1.00**	**1.00**	**0.42**
C–Models for Excretion N:P	AICc	∆AICc	Rel. Lik.	w_*i*_	r^2^
No effects	187.0	15.4	0.00	0.00	0.01
**Diet**	**171.6**	**0.0**	**1.00**	**0.50**	**0.28**
Predation	188.2	16.6	0.00	0.00	0.03
**Diet + Pred**	**171.6**	**0.0**	**1.00**	**0.50**	**0.31**

### Excretion rate vs. whole-body nutrients: Population means

**Fig 3:**
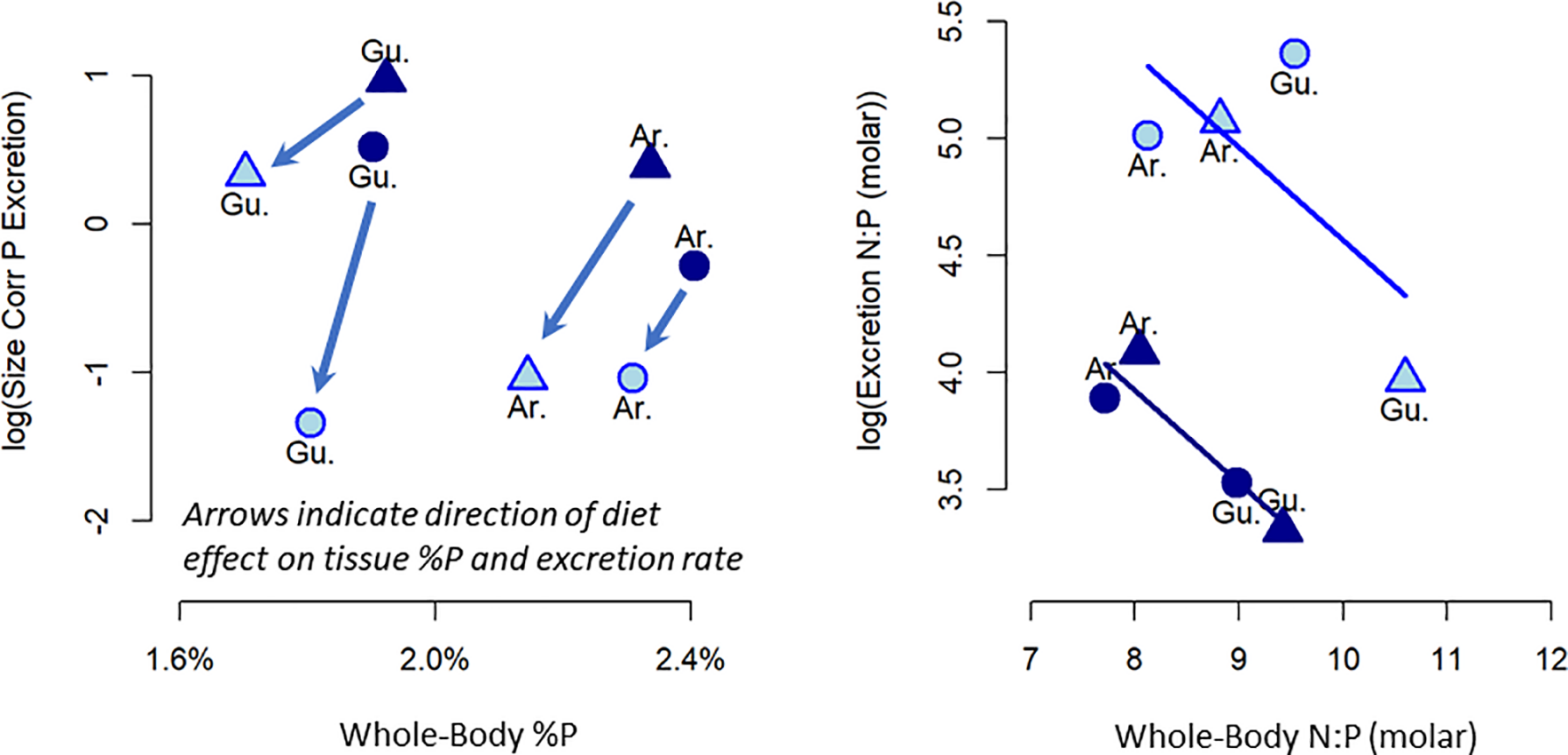
Size-corrected P excretion vs. whole-body P content (A) and excretion N:P vs. whole-body N:P (B). Symbols represent mean values for each population × diet treatment. Symbols shown here are high predation (“HPred”; triangles) and low predation (“LPred”; circles) guppies from the Guanapo (“Gu.”) and Aripo (“Ar.”) Rivers on the high-P diet (dark blue symbols and lines) and low-P diet (light blue symbols and lines). (A) For each population, low-P diet decreases both whole-body P and excretion P (arrows). (B) Lines represent best fit linear models for population means on the high-P diet (dark blue line) and the low-P diet (blue line). Population mean excretion N:P declines with population mean whole-body N:P content, but this relationship is confounded by the positive effect of diet on both whole-body and excretion N:P.

We modeled population-mean excretion N:P as a function of population-mean whole-body N:P and found no correlation (Table 2; slope = -0.06, slope standard error = 0.33, p = 0.85). However, the low-P diet increased both whole-body and excretion N:P (by reducing P in both tissue and excretion; Fig 3A). A model for excretion N:P with both diet and whole-body N:P indicated a significant negative correlation between a population’s whole-body N:P and its excretion N:P, after accounting for the effect of diet (Table 2; Fig 3; slope = -0.40, slope standard error = 0.16, p = 0.05). There was no significant correlation between N excretion and whole-body N (t = 0.393, p = 0.71) and there was a marginal, negative correlation between whole-body and excretion P after accounting for diet (t = -2.2, p = 0.08).

**Table 2:** Models for population-mean excretion N:P (log-transformed) versus population-mean whole-body N:P. Tables show key statistics on model fits and regression slope without diet (left) and with diet (right). Without accounting for diet, there is no correlation between whole-body N:P and excretion N:P. After accounting for the effect of diet, the overall model fit improves and a significant, negative correlation between whole-body N:P and excretion N:P is evident.

	Models For Excretion N:P vs. Whole-body N:P	
	Whole-body N:P Only	Whole-body N:P + Diet
AICc	29.3	24.0
R^2^	0.01	0.84
Adjusted R^2^	-0.16	0.78
Slope*	-0.06	-0.40
Slope Standard Error	0.33	0.16
Slope p value	0.85	0.05

*Excretion N:P vs. Whole-body N:P

### Survival and growth

We recorded no mortality events, and all guppies gained weight and length during the experiment. There were no treatment or population effects on growth rate (likelihood ratio (LR) tests, population: χ^2^ = 0.57, df = 1, p = 0.45; diet: χ^2^ = 0.19, df = 1, p = 0.66). At the conclusion of the experiment, guppies in different treatment groups were not significantly different in weight (df = 54, F = 0.34, p = 0.79). HP guppies did not differ from LP (HP mean = 0.36 g, LP mean = 0.38g, ± S.E. = 0.03g), and high-phosphorus diet guppies did not differ from low phosphorus diet guppies (High-P diet mean = 0.39g, Low-P diet mean = 0.36g, ± S.E. = 0.03g).

## Discussion

In this study, we assessed whether flexible homeostasis in whole-body nutrients could obscure the patterns predicted by mass balance models. We compared the whole-body and excretion nutrients of genetically diverged guppy populations when reared on diets with different nutrient contents. Our results show flexible homeostasis in guppy whole-body nutrients under variable diet quality, and this flexible homeostasis obscured the negative correlation between whole-body and excretion nutrients that is predicted by mass balance models. Below, we consider the implications of these results for mass balance models and their relevance to field surveys of variation in excretion.

Our results both support and contradict mass balance models, which predict nutrient excretion should be positively related to the supply of dietary nutrients and negatively to whole-body nutrients. Guppy P excretion was higher on the high-P diet. (Fig 2B). This result is consistent with a recent meta-analysis correlating diet and excretion stoichiometry in diverse fishes in aquaculture [18], suggesting this effect may be general among fish with variable quality diets. Mass balance also predicts that excretion N:P should be negatively related to whole-body N:P. Looking across all guppies in this study, we found no such overall correlation between N:P excretion and whole-body N:P. On the surface, this result is consistent with a recent survey of excretion rates among dozens of fish species [14], which found no evidence for a correlation between whole-body N:P and excretion N:P. Further review of our results, however, suggests that our lack of a negative correlation between whole-body and excretion N:P stems from the confounding influence of diet quality on both whole-body and excretion nutrients.

In our experiment, whole-body P was correlated with diet quality. Guppies on the low-P diet had lower whole-body %P (Fig 1), higher whole-body N:P (Fig 3), and higher whole-body C:P (Table B in S2 Table) providing evidence for dietary effects on whole-body P content. Such plasticity has been noted in some (but not all) field studies [12,46–48] and is likely universal in fish. Fish can deposit excess dietary P into skeletal stores [19,20,49], causing increases in skeletal %P and whole-body %P on high-P diets. This “luxury” deposition, however does not entirely account for all of the excess dietary P, so high-P diet guppies also excrete more P. Whole-body and excretion P are thus positively related, as evidenced by comparing the High-P and Low-P diet treatments for each of the four populations in Fig 3A. In each of the four populations, the Low-P diet treatment is below (lower P excretion) and to the left (lower whole-body P) of the High-P diet treatment.

After accounting for this diet-driven plasticity in both whole-body and excretion P, we detected a negative correlation between whole-body and excretion P among populations that may be attributed to genetics (HP vs. LP are genetically distinct in each of two rivers) or ontogeny (ages varied in Guanapo and Aripo). As predicted by mass balance models, guppy populations with the highest whole-body N:P had the lowest excretion N:P. Whole-body N:P and diet explained 84% of total variation in P excretion among guppy populations and treatments (Table 2). These results suggest that differences in whole-body and excretion P among genetically diverged populations may follow standard paradigms of mass balance models, but only if diet quality can be accurately assessed. This negative correlation reflects the homeostatic, mass-balance constraint: divergence in each population’s body plan alters demand for P to build incremental whole-body and thus changes the availability of excess P for excretion.

This result reaffirms the importance of characterizing diet quality when assessing stoichiometric patterns in excretion rate, a substantial challenge for field-based surveys [50]. Proxies for diet quality like trophic position [14] or trophic guild [51] can offer some insight into diet quality, but may miss variation in the specific nutrient (like P) that underlies much plastic variation in both whole-body and excretion P. Because it can be difficult to assess diet quality in the field, researchers may continue to struggle to find negative relationships between whole-body and excretion nutrients in field studies (*e*.*g*., Allgeier et al. 2015), even if this mechanism occurs widely among fish.

Yet diet and whole-body nutrient content alone are unlikely to be sufficient to describe variation in excretion in every situation. For instance, variation in excretion has previously been shown to be linked to body condition [52], nutrient acquisition and assimilation [15], or predation risk [53], among other factors. Our results support that diet quality and whole-body nutrient demand can both affect nutrient excretion rates, but it is likely that many studies will not find these factors due to other confounding influences, such as those mentioned above. The primary factors affecting excretion are likely to be context-dependent, and, here, we identify two factors that are potentially important in some, but not all, circumstances.

Notably, we did not detect any association between whole-body N and excretion N. This lack of relationship between whole-body and excretion N may stem from N’s important role in energy metabolism. Fish use amino acids for energy [54], and most N excretion in fish results from release of ammonium as a toxic byproduct of catabolism of amino acids for energy-yielding reactions [55,56]. P excretion in fish, on the other hand, is under homeostatic control and largely occurs via urinary pathways [57]. Thus, N excretion is much more likely to reflect processes related to energy metabolism, independent of whole-body N content, whereas P excretion will reflect the balance of tissue composition related to tissue growth and maintenance.

While we have focused on nutrient excretion as the mechanism by which organisms regulate homeostasis, variation in assimilation can also play a substantial role in mass balance. Organisms can increase assimilation of limiting nutrients [51] or decrease assimilation of nutrients present in excess [58]. Such compensatory mechanisms, however, would be expected to dampen the dietary influence on excretion rate. For example, a guppy on a high P diet might assimilate a smaller fraction of dietary P, decreasing its supply of P available for excretion on a high P diet. In turn, a guppy on a low P diet might assimilate a larger fraction of its dietary P, increasing the supply of P available for excretion on a low P diet. Supporting this inference is that fact that guppies from habitats with low-P foods have traits associated with higher P assimilation—longer guts and higher intestinal phosphatase activity [24,59]. We are unable to assess assimilation efficiency in this study, due to difficulties collecting egested material. Nonetheless, we posit that we detected differences in excretion on the high P and low P diets in spite of confounding effects of assimilation efficiency, not because of them.

### Genetic divergence of whole-body nutrient content and excretion along a replicated ecological gradient

Similar to field surveys [6,25,47,48], we found significant variation in whole-body and excretion nutrients of guppy populations from different environments (Figs 1 and 2). In this study, we used standardized laboratory conditions and genetically diverged populations [60] to attribute this variation to local genetic differentiation. Guppies from HPred sites tended to have higher excretion rates than guppies from LPred sites (for N and P; Fig 2). This result mirrors measures from mesocosm and field studies [25]. The persistence of higher N excretion after 1–2 generations of lab breeding, moreover, suggests genetic divergence contributes to the HPred and LPred differences observed in these field studies, and likely compounds diet-driven differences in N and P excretion.

Our lab study cannot definitively provide an adaptive significance for the genetic differences in excretion that we observed, though selection by diet quality provides an intriguing possibility. In nature, HPred guppies consume higher nutrient content foods than LPred [23]. Access to higher nutrient resources may relax selection on HPred guppies for nutrient efficiency, leading to higher nutrient losses via excretion (and thus higher excretion rates). But other traits, such as growth rate [30,61], behavior [62,63], metabolism [30,64], or morphology [65], also differ between HPred and LPred guppies and could affect the excretion of HPred and LPred populations. The influence of selection on these traits thus cannot be ruled out. Nonetheless, our data suggest that genetic differences among HPred and LPred populations are related to differences in excretion rates. Given the impacts of excretion rates on ecosystem function, our results thus provide a potential bridge between selection on organism traits and ecosystem function that warrants further investigation[28].

### Conclusion

While previous studies have varied in their ability to detect the patterns in excretion rate predicted by mass balance models, we suggest that this may relate to the confounding influences of diet and genetic differentiation. After accounting for the parallel effects of diet on whole-body and excretion P, we found the negative relationship between whole-body and excretion P predicted by mass balance. This pattern would not be evident in field studies that will struggle to accurately capture diet quality. Quantifying genetic divergence, phenotypic plasticity, and food nutrient density as causes of variation in whole-body nutrient content and excretion will remain central to understanding how environmental change will affect nutrient excretion by important consumers across diverse ecosystems.

## Supporting information

S1 Table Dietary phosphorus requirements. List of ingredients used to make guppy diets used in this study.
(DOCX)

S2 Table Detailed statistical results. Series of tables showing specific model comparisons used to support inferences made in this study.
(DOCX)

S3 Table River-specific analysis. Series of tables showing specific statistical results, separated by river.
(DOCX)

S1 Data Supporting information 1 (CSV). (CSV)
