# Supplementary material for: The influence of dietary and whole-body nutrient content on the excretion of a vertebrate consumer
Source: PLoS One. 2017 Nov 27;12(11):e0187931. doi: 10.1371/journal.pone.0187931 (PMC5703491; doi:10.1371/journal.pone.0187931)
Supplement: S1 Table — List of ingredients used to make guppy diets used in this study. (DOCX) [file pone.0187931.s001.docx]

**S1 Table**

**S1 Table:** Diet formulation for high and low phosphorus diets, modified from Shim and Ho (1989). Total diet %P was 0.26% for the Low P and 0.81% for the High P diet. The total P content of the low and high P diets is below and above, respectively, the estimate of P limitation derived by Shim & Ho for domestic guppies. Sieved sand was added to the diet as an inert replacement, to keep the percent of all other diet items in the diet the same despite adding additional phosphorus to the high phosphorus diet.

|  | Low P Diet | High P Diet |  |
| --- | --- | --- | --- |
| Ingredient | Amount added  (g/ kg diet) | Amount added  (g/ kg diet) | Ingredient P Content  (g / kg item) |
| Vitamin Free Casein | 351.2 | 351.2 | 7.5 |
| Starch | 150.5 | 150.5 | - |
| Dextrin | 141.6 | 141.6 | - |
| Gelatin | 78.1 | 78.1 | 0.2 |
| Vegetable oil | 50.0 | 50.0 | - |
| Fish oil | 50.1 | 50.1 | - |
| Cellulose - FCC | 10.0 | 10.0 | - |
| Chlorine Chloride | 5.0 | 5.0 | - |
| Ascorbic Acid | 1.2 | 1.2 | - |
| DL-methionine | 2.0 | 2.0 | - |
| L-tryptophan | 1.0 | 1.0 | - |
| Betaine | 10.0 | 10.0 | - |
| Thiamine | 0.0 | 0.0 | - |
| Vitamin Premix | 5.0 | 5.0 | - |
| Dibasic Calcium Phosphate | 0.0 | 30.1 | 182.1 |
| Sieved sand | 42.1 | 12.0 | - |
| Mineral Mixture | 102.0 | 102.0 | - |

The composition of the mineral mix was as below, all values in g / kg of mineral mix:

MgSO_4_: 30.0; FeSO_4_×7H_2_0: 5.0; NaHCO_3_: 88.8; ZnCO_3_: 1.5; CuSO_4_×5H_2_0: 0.3; MnSO_4_×H_2_0: 3.5; KIO_3_: 0.1; CoCL_2_×6H_2_0: 0.02; Na_2_MoO_4_×2H_2_0: 0.14; Alpha-cellulose: 727.9; CaCO_3_: 142.8
