# Supplementary material for: The influence of dietary and whole-body nutrient content on the excretion of a vertebrate consumer
Source: PLoS One. 2017 Nov 27;12(11):e0187931. doi: 10.1371/journal.pone.0187931 (PMC5703491; doi:10.1371/journal.pone.0187931)
Supplement: S2 Table — Series of tables showing specific model comparisons used to support inferences made in this study. (DOCX) [file pone.0187931.s002.docx]

**S2 Table**

**S2, Table A:** Models of %C, %N, and %P with different combinations of diet treatment and ancestral predation effects. All models include ‘river’ as a random effect, to account for differences between guppies from the Aripo and Guanapo Rivers in the age, rearing environment, and background genetics. Models with ΔAICc <2.0 are formatted bold, indicating strong support. Values of r^2^ reflect conditional r^2^, the percent of variance explained by all factors in the model.

*Whole-body C content* was best explained by a model with only a diet effect, though removing the diet term did not significantly reduce model explanatory power (likelihood ratio test (LRT); df = 1, χ^2^ = 2.80, p = 0.09). Guppies on the low P diet averaged 0.7% ± 0.4% higher %C than high P diet guppies.

| Models for Variation in %C | | | | |  |
| --- | --- | --- | --- | --- | --- |
|  | **AICc** | **∆AICc** | **Rel. Lik.** | **w*_i_*** | **r^2^** |
| **No effects** | **-311.9** | **0.5** | **0.37** | **0.27** | **0.27** |
| **Diet** | **-312.4** | **0.0** | **1.00** | **0.72** | **0.31** |
| Predation | -309.6 | 2.8 | 0.00 | 0.00 | 0.27 |
| Diet + Pred | -310 | 2.4 | 0.01 | 0.01 | 0.32 |

*Whole-body N content* was best explained by a model with only a predation environment effect, though removing the predation term only marginally reduced model explanatory power (LRT: df = 1, χ^2^ = 3.78, p = 0.05). HPred guppies averaged 0.2% ± 0.1% higher whole-body N content than LPred guppies.

| Models for Variation in %N | | | | |  |
| --- | --- | --- | --- | --- | --- |
|  | AICc | ∆AICc | Rel. Lik. | w*_i_* | r^2^ |
| **No effects** | **-447.5** | **1.4** | **0.06** | **0.06** | **0.30** |
| Diet | -445.5 | 3.4 | 0.00 | 0.00 | 0.30 |
| **Predation** | **-448.9** | **0.0** | **1.00** | **0.93** | **0.35** |
| Diet + Pred | -446.7 | 2.2 | 0.01 | 0.01 | 0.35 |

*Whole-body P content* was best explained by a model with only a diet term. Removing the diet term significantly reduced model explanatory power (LRT: df = 1, χ2 = 4.42, p = 0.04), and adding the predation term did not significantly increase model explanatory power (LRT: df = 1, χ^2^ = 1.19, p = 0.28). Guppies on the high P diet had 12% higher whole-body P stocks than those on the low P diet (high P: 2.2%; low P: 1.9%).

| Models for Variation in %P | | | | |  |
| --- | --- | --- | --- | --- | --- |
|  | AICc | ∆AICc | Rel. Lik. | w*_i_* | r^2^ |
| No effects | -503.7 | 2.1 | 0.01 | 0.01 | 0.39 |
| **Diet** | **-505.8** | **0.0** | **1.00** | **0.91** | **0.44** |
| Predation | -502.8 | 3.0 | 0.00 | 0.00 | 0.40 |
| **Diet + Pred** | **-504.6** | **1.2** | **0.09** | **0.08** | **0.45** |

**S2, Table B:** Models of C:N, C:P, and N:P with different combinations of diet treatment and ancestral predation effects. All models include ‘river’ as a random effect, to account for differences between guppies from the Aripo and Guanapo Rivers in the age, rearing environment, and background genetics. Models with ΔAICc <2.0 are formatted bold, indicating strong support.

*Whole-body C:N* (S2; Table 2) was best explained by a model with no fixed effects. Adding the predation term did not significantly increase model explanatory power (LRT: df = 1, χ^2^ = 1.96, p = 0.16).

| Models for Variation in C:N | | | | |  |
| --- | --- | --- | --- | --- | --- |
|  | AICc | ∆AICc | Rel. Lik. | w*_i_* | r^2^ |
| **No effects** | **115.3** | **0.0** | **1.00** | **0.64** | **0.34** |
| Diet | 117.5 | 2.2 | 0.01 | 0.01 | 0.34 |
| **Predation** | **115.6** | **0.3** | **0.55** | **0.35** | **0.37** |
| Diet + Pred | 117.9 | 2.6 | 0.01 | 0.01 | 0.37 |
|  |  |  |  |  |  |

*Whole-body C:P* was best explained by a model with only a diet effect. Adding a predation effect did not increase model explanatory power (LRT: df = 1, χ^2^ = 1.30, p = 0.26), but removing the diet term did significantly reduce model explanatory power (LRT: df = 1, χ^2^ = 4.54, p = 0.03). Low P diet guppies averaged 9% higher C:P than high P diet guppies (69.9 vs. 64.4).

| Models for Variation in C:P | | | | |  |
| --- | --- | --- | --- | --- | --- |
|  | AICc | ∆AICc | Rel. Lik. | w*_i_* | r^2^ |
| No effects | 457.2 | 2.2 | 0.01 | 0.01 | 0.38 |
| **Diet** | **455.0** | **0.0** | **1.00** | **0.89** | **0.44** |
| Predation | 458.0 | 3.0 | 0.00 | 0.00 | 0.40 |
| **Diet + Pred** | **456.1** | **1.1** | **0.11** | **0.10** | **0.45** |
|  |  |  |  |  |  |

*Whole-body N:P* was best explained by a model with both a diet and predation term. Removing the predation term only marginally reduced model explanatory power (LRT: df = 1, χ^2^ = 3.62, p = 0.06), but removing the diet term significantly reduced model explanatory power (LRT: df = 1, χ^2^ = 4.38, p = 0.04). Guppies on the low P diet had 8% higher N:P than those on the high P diet (high P N:P = 8.6; low P N:P = 9.3).

| Models for Variation in N:P | | | | |  |  |
| --- | --- | --- | --- | --- | --- | --- |
|  | AICc | ∆AICc | Rel. Lik. | w*_i_* | r^2^ |  |
| No effects | 211.9 | 3.6 | 0.00 | 0.00 | 0.19 |  |
| **Diet** | **209.5** | **1.2** | **0.09** | **0.08** | **0.26** |  |
| Predation | 210.3 | 2.0 | 0.02 | 0.02 | 0.25 |  |
| **Diet + Pred** | **208.3** | **0.0** | **1.00** | **0.90** | **0.31** |  |
