# Supplementary material for: The influence of dietary and whole-body nutrient content on the excretion of a vertebrate consumer
Source: PLoS One. 2017 Nov 27;12(11):e0187931. doi: 10.1371/journal.pone.0187931 (PMC5703491; doi:10.1371/journal.pone.0187931)
Supplement: S3 Table — Series of tables showing specific statistical results, separated by river. (DOCX) [file pone.0187931.s003.docx]

**S3, Table 1:** Models of %C, %N, and %P with different combinations of diet treatment and ancestral predation effects for the Aripo (left) and Guanapo (right) Rivers. Models with ΔAICc <2.0 are formatted bold, indicating some support for that model.

*% C*: In both the Aripo and Guanapo, a model with diet received substantial support. The diet effect was marginally significant in the Aripo River (p = 0.08) and not significant in the Guanapo River (p = 0.45). In both cases, whole-body %C was higher with the low P than high P diet (Guanapo: 51.8% High Phos vs. 52.2% Low Phos; Aripo: 49.6% High Phos vs. 50.5% Low Phos).

| **Models for Variation in %C** | | | | |  |  |  |  |  |  |  |
| --- | --- | --- | --- | --- | --- | --- | --- | --- | --- | --- | --- |
|  |  |  | **Aripo** |  |  |  | **Guanapo** | | | | |
|  | **AICc** | **∆AICc** | **Rel. Lik.** | **w*_i_*** | **r^2^** |  | **AICc** | **∆AICc** | **Rel. Lik.** | **w*_i_*** | **r^2^** |
| **No effects** | **-144.5** | **0.6** | **0.30** | **0.23** | **0.00** |  | **-172.2** | **0.0** | **1.00** | **0.96** | **0.00** |
| **Diet** | **-145.1** | **0.0** | **1.00** | **0.76** | **0.12** |  | **-170.4** | **1.8** | **0.03** | **0.03** | **0.02** |
| Predation | -142.0 | 3.1 | 0.00 | 0.00 | 0.00 |  | -170.1 | 2.1 | 0.01 | 0.01 | 0.01 |
| Diet + Pred | -142.5 | 2.6 | 0.01 | 0.01 | 0.12 |  | -168.1 | 4.1 | 0.00 | 0.00 | 0.03 |

*%N*: In both the Aripo and Guanapo, a model with Predation received substantial support. In the Aripo, this effect was not significant (p = 0.44) but it was marginally significant in the Guanapo (p = 0.08). Whole-body %N was 0.1% lower in the Aripo (8.5% vs. 8.4%) and 0.3% lower in the Guanapo (7.9% vs. 7.6%).

| **Models for Variation in %N** | | | | |  |  |  |  |  |  |  |
| --- | --- | --- | --- | --- | --- | --- | --- | --- | --- | --- | --- |
|  |  |  | **Aripo** |  |  |  | **Guanapo** | | | | |
|  | **AICc** | **∆AICc** | **Rel. Lik.** | **w*_i_*** | **r^2^** |  | **AICc** | **∆AICc** | **Rel. Lik.** | **w*_i_*** | **r^2^** |
| **No effects** | **-214.2** | **0.0** | **1.00** | **0.97** | **0.00** |  | **-241.6** | **1.0** | **0.14** | **0.12** | **0.00** |
| Diet | -211.6 | 2.6 | 0.01 | 0.01 | 0.00 |  | -239.4 | 3.2 | 0.00 | 0.00 | 0.01 |
| **Predation** | **-212.3** | **1.9** | **0.02** | **0.02** | **0.02** |  | **-242.6** | **0.0** | **1.00** | **0.87** | **0.10** |
| Diet + Pred | -209.5 | 4.7 | 0.00 | 0.00 | 0.03 |  | -240.1 | 2.5 | 0.01 | 0.01 | 0.11 |

*%P*: In both the Aripo and Guanapo, a model with diet received substantial support but the diet effect was not significant in either river, when analyzed independently (p = 0.16 in Aripo and p = 0.13 in Guanapo). Low Phos diet guppies had lower whole-body %P in both rivers (1.7% vs. 1.9% in Guanapo; 2.2% vs. 2.3% in Aripo).

| **Models for Variation in %P** | | | | |  |  |  |  |  |  |  |
| --- | --- | --- | --- | --- | --- | --- | --- | --- | --- | --- | --- |
|  |  |  | **Aripo** |  |  |  | **Guanapo** | | | | |
|  | **AICc** | **∆AICc** | **Rel. Lik.** | **w*_i_*** | **r^2^** |  | **AICc** | **∆AICc** | **Rel. Lik.** | **w*_i_*** | **r^2^** |
| **No effects** | **-230.6** | **0.0** | **1.00** | **0.61** | **0.00** |  | **-278.8** | **0.1** | **0.82** | **0.45** | **0.00** |
| **Diet** | **-230.2** | **0.4** | **0.45** | **0.27** | **0.08** |  | **-278.9** | **0.0** | **1.00** | **0.54** | **0.07** |
| Predation | **-229.7** | **0.9** | **0.17** | **0.10** | **0.06** |  | -276.6 | 2.3 | 0.01 | 0.01 | 0.01 |
| Diet + Pred | **-228.9** | **1.7** | **0.03** | **0.02** | **0.13** |  | -276.4 | 2.5 | 0.01 | 0.01 | 0.08 |

**S3, Table 2:** Models of C:N, C:P, and N:P with different combinations of diet treatment and ancestral predation effects. Models with ΔAICc <2.0 are formatted bold, indicating support.

*C:N*: In both the Aripo and Guanapo guppies, a model with no effects received the most support. Models with a predation term received the next most support, but this term was not significant in either river. In both cases, LP guppies had slightly higher C:N.

| **Models for Variation in C:N** | | | | |  |  |  |  |  |  |  |
| --- | --- | --- | --- | --- | --- | --- | --- | --- | --- | --- | --- |
|  |  |  | **Aripo** |  |  |  | **Guanapo** | | | | |
|  | **AICc** | **∆AICc** | **Rel. Lik.** | **w*_i_*** | **r^2^** |  | **AICc** | **∆AICc** | **Rel. Lik.** | **w*_i_*** | **r^2^** |
| No effects | **35.4** | **0.0** | **1.00** | **0.98** | **0.00** |  | **69.8** | **0.0** | **1.00** | **0.83** | **0.00** |
| Diet | 37.6 | 2.2 | 0.01 | 0.01 | 0.02 |  | 72.2 | 2.4 | 0.01 | 0.01 | 0.00 |
| Predation | 37.5 | 2.1 | 0.01 | 0.01 | 0.02 |  | **70.6** | **0.8** | **0.20** | **0.17** | **0.05** |
| Diet + Pred | 39.9 | 4.5 | 0.00 | 0.00 | 0.03 |  | 73.3 | 3.5 | 0.00 | 0.00 | 0.05 |

*C:P*: In both the Aripo and Guanapo, models with a ‘diet’ term received the most support. In the Aripo, this diet effect was marginally significant (p = 0.07), and it was not significant in the Guanapo (p = 0.15). In both rivers, C:P was higher on the low Phos diet (Guanapo: 72 on high P vs. 78 on low P; Aripo: 54 on the high P vs. 59 on the low P).

| **Models for Variation in C:P** | | | | |  |  |  |  |  |  |  |
| --- | --- | --- | --- | --- | --- | --- | --- | --- | --- | --- | --- |
|  |  |  | **Aripo** |  |  |  | **Guanapo** | | | | |
|  | **AICc** | **∆AICc** | **Rel. Lik.** | **w*_i_*** | **r^2^** |  | **AICc** | **∆AICc** | **Rel. Lik.** | **w*_i_*** | **r^2^** |
| **No effects** | **182.5** | **1.0** | **0.14** | **0.12** | **0.00** |  | **260.5** | **0.0** | **1.00** | **0.58** | **0.00** |
| **Diet** | **181.5** | **0.0** | **1.00** | **0.85** | **0.13** |  | **260.7** | **0.2** | **0.67** | **0.39** | **0.07** |
| Predation | 183.9 | 2.4 | 0.01 | 0.01 | 0.04 |  | **262.2** | **1.7** | **0.03** | **0.02** | **0.02** |
| Diet + Pred | **183.4** | **1.9** | **0.02** | **0.02** | **0.16** |  | 262.7 | 2.2 | 0.01 | 0.01 | 0.09 |

*N:P*: In both the Aripo and Guanapo, many models received substantial support. In the Aripo, the diet effect was marginally significant and the predation effect was not significant (p = 0.8 and p = 0.11, respectively). In the Guanapo, neither effect was significant (diet: p = 0.15; predation: p = 0.20). LP guppies had lower N:P and low Phos diet guppies had higher N:P.

| **Models for Variation in N:P** | | | | |  |  |  |  |  |  |  |
| --- | --- | --- | --- | --- | --- | --- | --- | --- | --- | --- | --- |
|  |  |  | **Aripo** |  |  |  | **Guanapo** | | | | |
|  | **AICc** | **∆AICc** | **Rel. Lik.** | **w*_i_*** | **r^2^** |  | **AICc** | **∆AICc** | **Rel. Lik.** | **w*_i_*** | **r^2^** |
| **No effects** | **70.0** | **1.2** | **0.09** | **0.04** | **0.00** |  | **127.1** | **0.0** | **1.00** | **0.37** | **0.00** |
| **Diet** | **68.9** | **0.1** | **0.82** | **0.38** | **0.13** |  | **127.1** | **0.0** | **1.00** | **0.37** | **0.07** |
| **Predation** | **69.5** | **0.7** | **0.25** | **0.12** | **0.11** |  | **127.5** | **0.4** | **0.45** | **0.17** | **0.06** |
| **Diet + Pred** | **68.8** | **0.0** | **1.00** | **0.46** | **0.23** |  | **127.8** | **0.7** | **0.25** | **0.09** | **0.12** |

**S3, Table 3:** Models of N and P excretion with different combinations of diet treatment and ancestral predation effects. Models with ΔAICc <2.0 are formatted bold, indicating support.

*N Excretion (Size-Corrected)*: In both the Aripo and Guanapo, models with a predation term received substantial support. The predation term was significant in the Guanapo (p = 0.02) and not significant in the Aripo (p = 0.18). Guppies from HPred environments had higher N excretion (Aripo: 90 vs. 65 µg hr^-1^ g^3/4^; Guanapo: 91 vs. 65 µg hr^-1^ g^3/4^).

| **Models for Variation in Size-Corrected N Excretion** | | | | | | | | | | | |
| --- | --- | --- | --- | --- | --- | --- | --- | --- | --- | --- | --- |
|  |  |  | **Aripo** |  |  |  | **Guanapo** | | | | |
|  | **AICc** | **∆AICc** | **Rel. Lik.** | **w*_i_*** | **r^2^** |  | **AICc** | **∆AICc** | **Rel. Lik.** | **w*_i_*** | **r^2^** |
| **No effects** | **277.7** | **0.0** | **1.00** | **0.76** | **0.00** |  | 319.4 | 3.2 | 0.00 | 0.00 | 0.00 |
| Diet | 280.3 | 2.6 | 0.01 | 0.01 | 0.00 |  | 321.8 | 5.6 | 0.00 | 0.00 | 0.00 |
| **Predation** | **278.3** | **0.6** | **0.30** | **0.23** | **0.07** |  | **316.2** | **0.0** | **1.00** | **1.00** | **0.16** |
| Diet + Pred | 281.1 | 3.4 | 0.00 | 0.00 | 0.07 |  | 318.9 | 2.7 | 0.00 | 0.00 | 0.16 |

*P Excretion (Size-Corrected)*: In both rivers, models with at least a diet term received support. In both rivers, this diet term was significant (Aripo: p = 0.01; Guanapo: p = 0.001). In Guanapo, the predation term was significant (p = 0.01), but not in the Aripo (p = 0.38). In both rivers, high phos diet and HPred guppies had higher P excretion (Aripo: High Phos = 3.3 µg hr^-1^ g^-3/4^ Low Phos = 1.29 µg hr^-1^ g^-3/4^; Guanapo: High Phos = 6.4 µg hr^-1^ g^-3/4^ Low Phos = 2.6 µg hr^-1^ g^-3/4^)

| **Models for Variation in Size-Corrected P Excretion** | | | | | | | | | | | |
| --- | --- | --- | --- | --- | --- | --- | --- | --- | --- | --- | --- |
|  |  |  | **Aripo** |  |  |  | **Guanapo** | | | | |
|  | **AICc** | **∆AICc** | **Rel. Lik.** | **w*_i_*** | **r^2^** |  | **AICc** | **∆AICc** | **Rel. Lik.** | **w*_i_*** | **r^2^** |
| No effects | 76.5 | 6.0 | 0.00 | 0.00 | 0.00 |  | 104.8 | 17.2 | 0.00 | 0.00 | 0.00 |
| **Diet** | **70.5** | **0.0** | **1.00** | **0.98** | **0.28** |  | 97.1 | 9.5 | 0.00 | 0.00 | 0.27 |
| Predation | 78.8 | 8.3 | 0.00 | 0.00 | 0.01 |  | 100.2 | 12.6 | 0.00 | 0.00 | 0.20 |
| **Diet + Pred** | **72.4** | **1.9** | **0.02** | **0.02** | **0.30** |  | **87.6** | **0.0** | **1.00** | **1.00** | **0.50** |

*Excretion N:P (log-corrected)*: In both rivers, models with at least a diet term received strong support. In both the Aripo and Guanapo, the diet term was significant (Aripo: p = 0.02; Guanapo: p < 0.001). In the Guanapo, the predation term was significant (p = 0.01), but it was not in the Aripo (p = 0.78). In both rivers, high phos diet guppies had lower excretion N:P.

| **Models for Variation in Excretion N:P (log transformed)** | | | | | | | | | | | |
| --- | --- | --- | --- | --- | --- | --- | --- | --- | --- | --- | --- |
|  |  |  | **Aripo** |  |  |  | **Guanapo** | | | | |
|  | **AICc** | **∆AICc** | **Rel. Lik.** | **w*_i_*** | **r^2^** |  | **AICc** | **∆AICc** | **Rel. Lik.** | **w*_i_*** | **r^2^** |
| No effects | 86.3 | 3.6 | 0.00 | 0.00 | 0.00 |  | 100.4 | 14.4 | 0.00 | 0.00 | 0.00 |
| **Diet** | **82.7** | **0.0** | **1.00** | **1.00** | **0.21** |  | 90.5 | 4.5 | 0.00 | 0.00 | 0.32 |
| Predation | 88.6 | 5.9 | 0.00 | 0.00 | 0.01 |  | 99.1 | 13.1 | 0.00 | 0.00 | 0.11 |
| Diet + Pred | 85.4 | 2.7 | 0.00 | 0.00 | 0.21 |  | **86** | **0.0** | **1.00** | **1.00** | **0.46** |
